# Effect of six weeks ubiquinol supplementation on mitochondrial respiratory function and exercise capacity in healthy males

**DOI:** 10.1007/s00421-026-06275-w

**Published:** 2026-06-06

**Authors:** Jarred P. Acton, Nehal S. Alsharif, Jack W. Bond, Stuart P. Cocksedge, Abbie G. Mclellan, Donald L. Peden, Mark P. Funnell, Hannah F. Dugdale, Lewis J. James, Richard A. Ferguson, Tom Clifford, Stephen J. Bailey

**Affiliations:** 1 School of Sport, Exercise and Health Sciences, Loughborough University, Loughborough, UK; 2 Department of Clinical Nutrition, Faculty of Applied Medical Science, King Abdulaziz University, Jeddah, Saudi Arabia; 3 Centre for Physical Activity, Sport and Exercise Sciences, Coventry University, Coventry, UK; 4 David Greenfield Human Physiology Unit, School of Life Sciences, Nottingham NIHR Biomedical Research Centre, University of Nottingham, Nottingham, UK; 5 NIHR Applied Research Collaboration East Midlands, Diabetes Research Centre, University of Leicester, Leicester, UK; 6 NIHR Leicester Biomedical Research Centre, University Hospitals of Leicester NHS Trust and the University of Leicester, Leicester, UK

**Keywords:** Mitochondrial respiratory efficiency, Exercise performance, Coenzyme Q10, V̇O_2_ kinetics, Dietary supplement

## Abstract

Coenzyme Q10 (CoQ_10_) is an integral component of the mitochondrial electron transfer system. Most studies have administered the oxidised form of CoQ_10_ (ubiquinone) and observed no effects on mitochondrial respiratory function or endurance exercise performance. The reduced form of CoQ_10_, ubiquinol (UQH_2_), has greater bioavailability than ubiquinone, but the effects of UQH_2_ supplementation on mitochondrial respiratory function and exercise capacity are unclear. Fifty-four healthy, recreationally active males were randomised to receive either 300 mg·day^− 1^ UQH_2_ or placebo (PLA) for 6 weeks in a double-blind independent-group design. Before and after the supplementation period, skeletal muscle mitochondrial respiration variables and protein content of the mitochondrial leak proteins, adenine nucleotide translocase1 + 2 (ANT1 + 2) and uncoupling protein-3 (UCP-3), were assessed. In addition, participants completed a severe-intensity cycle test to exhaustion to assess time to the limit of tolerance (T_Lim_) and oxygen uptake (V̇O_2_) kinetics. Compared to pre-supplementation and PLA, UQH_2_ supplementation increased plasma [CoQ_10_] (*P* < 0.05), and lowered inverse respiratory control ratio (Pre-PLA: 0.064 ± 0.034 vs. Post-PLA: 0.072 ± 0.026; Pre- UQH_2_: 0.073 ± 0.039 vs. Post-UQH_2_: 0.044 ± 0.019; *P* < 0.05), suggestive of improved oxidative phosphorylation coupling efficiency. There were no differences in ANT1 + 2 or UCP-3 protein content post-supplementation compared to pre-supplementation between groups (*P* > 0.05). End-exercise V̇O_2_, change in V̇O_2_ between 2 min and end-exercise, and T_Lim_ were not different between groups post-supplementation (*P* > 0.05). Six-weeks UQH_2_ supplementation increased plasma [CoQ_10_] and oxidative phosphorylation coupling efficiency, but did not alter mitochondrial leak proteins, T_Lim_ or V̇O_2_ kinetics during severe-intensity exercise in healthy, active males.

## Introduction

Oxidative phosphorylation of adenosine diphosphate (ADP) to adenosine triphosphate (ATP) in the mitochondrial electron transfer system (ETS) is the principal energy pathway used during endurance exercise (Bangsbo et al. 1990; Hargreaves and Spriet 2020). Coenzyme Q10 (CoQ_10_) is a lipophilic molecule ubiquitously expressed in cell membranes, including the inner mitochondrial membrane, where it functions as an essential component of the mitochondrial ETS by undergoing continuous redox cycles (Rauchová 2021). The oxidised form of CoQ_10_, ubiquinone, can be reduced to ubiquinol (UQH_2_) by electrons transferred from mitochondrial complex I (via NADH), mitochondrial complex II (via FADH_2_), glycerol 3-phosphate dehydrogenase (via glycolysis) and the electron-transferring flavoprotein (via fatty acid oxidation) (Gnaiger 2020). Subsequently, UQH_2_ functions as a mobile electron carrier in the inner mitochondrial membrane, where it is oxidised by mitochondrial complex III, with the resultant ubiquinone able to recommence the aforementioned redox cycle (Rich and Maréchal 2010). Consequently, CoQ_10_ availability is integral to preserve convergent electron transfer at the CoQ_10_ junction (Q junction) and subsequent electron transfer along the remaining mitochondrial respiratory complexes. Indeed, impaired mitochondrial respiration, alongside compromised exercise capacity, is a characteristic feature of individuals with mitochondrial myopathies that lower mitochondrial CoQ_10_ content. Importantly, these deficits can be abated with CoQ_10_ supplementation (Artuch et al. 2006; Barca et al. 2016; Lalani et al. 2005).

Given that effective skeletal muscle mitochondrial respiration is an important determinant of endurance exercise performance in healthy adults (Granata et al. 2016 b; Jacobs et al. 2011), there has been great interest in dietary CoQ_10_ supplements as potential ergogenic aids for endurance exercise (Fernandes et al. 2023; Sarmiento et al. 2016). Initial studies assessing the ergogenic potential of CoQ_10_ supplementation on endurance exercise performance administered CoQ_10_ as ubiquinone with most (Braun et al. 1991; Cooke et al. 2008; Laaksonen et al. 1995; Östman et al. 2012; Weston et al. 1997; Zhou et al. 2005), but not all (Gökbel et al. 2010; Ylikoski et al. 1997), reporting no ergogenic effects with ubiquinone supplementation. It is recognised that a limitation of CoQ_10_ supplementation is the low bioavailability after oral ingestion (Bhagavan and Chopra 2006) with studies reporting no significant increase in muscle [CoQ_10_] after ubiquinone supplementation (Cooke et al. 2008; Svensson et al. 1999). Importantly, however, the increase in plasma [CoQ10] after ubiquinone supplementation has been positively correlated with increased muscle [CoQ10], along with improved exercise performance (Cooke et al. 2008). Accordingly, supplementation with a more bioavailable form of CoQ10 may have greater potential to elicit ergogenic effects.

Initial studies administered CoQ10 as ubiquinone since UQH_2_ is ready oxidised to ubiquinone. In 2007, a stable form of UQH_2_ became commercially available (Hosoe et al. 2007). Compared to ubiquinone, UQH_2_ has demonstrated greater bioavailability following oral ingestion with large (*g* = 1.04; Langsjoen and Langsjoen 2014) to moderate ( *g =* 0.6; Zhang et al. 2018) effect sizes reported. As such, UQH_2_ supplementation may have greater potential to increase muscle [CoQ10] and elicit exercise performance improvements compared to ubiquinone supplementation (Cooke et al. 2008). However, despite potential for greater bioavailability and ergogenic effects, few studies have assessed the effect of UQH_2_ supplementation on exercise performance. Of the 3 studies conducted to date, 2 have reported no improvement in endurance exercise performance after 200–300 mg/day UQH_2_ supplementation for 4 weeks (Bloomer et al. 2012; Orlando et al. 2018). Conversely, 1 study reported a small increase (11.0%, *g* = 0.35) in the cycling power output at a fixed blood lactate concentration (4 mmol/L) compared to placebo (8.5%) in elite cyclists following 6 weeks of supplementaion with 300 mg/day of UQH_2_ (Alf et al. 2013). Therefore, supplementation with at least 300 mg/day UQH_2_ for a minimum of 6 weeks may be necessary to elicit an ergogenic effect during endurance exercise, but further research is required to verify this assertion.

Whilst it has been reported that 2 weeks supplementation with 200 mg/day UQH_2_ could improve platelet respiration in recovery from COVID-19 (Sumbalová et al. 2022), in the only study to have assessed the effect of UQH_2_ supplementation on mitochondrial respiration in human skeletal muscle, 6 weeks of supplementation with 200 mg/day UQH_2_ did not improve leak respiration, maximal ADP-stimulated respiration or maximal ETS activity (Pham et al. 2020). However, UQH_2_ supplementation did blunt the production of the reactive oxygen species (ROS), hydrogen peroxide (H_2_O_2_), during leak respiration (Pham et al. 2020). The ability of CoQ_10_ to undergo redox cycles is not only important for electron transfer in the mitochondrial ETS, but also allows UQH_2_ to donate electrons to and subsequently neutralise free radicals, which are highly reactive molecules that can damage cellular constituents, consequent to unpaired electrons in their outer orbital (Littarru and Tiano 2007). Although H_2_O_2_ is a non-radical ROS, it is synthesised in the mitochondria from the free radical ROS, superoxide (·O_2_^−^), via dismutation of two ·O_2_^−^ molecules in a reaction catalysed by superoxide dismutase 2 (Powers and Jackson 2008). Mitochondrial ROS production results from premature electron-leaks in the ETS, which contributes towards the uncoupling of mitochondrial O_2_ consumption at ETS complex IV from ATP synthesis at ETS complex V (Fang et al. 2020; Jastroch et al. 2010). Furthermore, ROS, or its derivatives such as the lipid peroxidation product, 4-hydroxy-2-nonenal (4-HNE), can activate mitochondrial leak proteins, adenine nucleotide translocase1 + 2 (ANT1 + 2) and uncoupling protein-3 (UCP-3) (Echtay et al. 2003; Klingenberg and Winkler 1985). In addition, mitochondria-derived ROS could contribute to mitochondrial leak respiration by damaging mitochondrial membranes via lipid peroxidation leading to a dissipation of the proton motive force (Nicholls and Ferguson 2013). Whilst Pham et al. (2020) did not observe lower mitochondrial leak respiration after UQH_2_ supplementation, despite lower H_2_O_2_ production, further research is required to assess whether supplementation with a higher dose of UQH_2_ can attenuate mitochondrial leak respiration and modulate other respiratory states.

The purpose of the current study was to assess the effects of 6 weeks supplementation with 300 mg∙day^− 1^ UQH_2_ on exercise capacity, assessed via time to the limit of tolerance (T_Lim_) during severe-intensity exercise, skeletal muscle mitochondrial respiration, and skeletal muscle protein content of ANT1 + 2 and UCP-3. It was hypothesised that UQH_2_ supplementation would increase T_Lim_ during severe-intensity exercise and lower skeletal muscle mitochondrial leak respiration and ANT1 + 2 and UCP-3 protein content leading to improved mitochondrial respiratory coupling efficiency.

## Methods

### Participants

Fifty-four healthy, recreationally active males volunteered to participate in this study. Participants did not have any current or historical neuromuscular, haematological, or musculoskeletal issues. Participants were non-smokers and were not using any dietary supplements or medication prior to, or during the study. Approval of the experimental procedures were granted by the Loughborough University Ethics Approvals (Human Participants) Sub-Committee. Participants received information of the experimental procedures, and associated risks and benefits, prior to providing written informed consent. Participants were instructed to arrive for laboratory visits in a rested state, at least 3 h post-prandial, having avoided strenuous physical activity and alcohol ingestion for 24 h and caffeine ingestion for 12 h. In the 24 h before the first experimental trial (visit 3), participants recorded their diet and any habitual low-intensity physical activity to enable replication in the 24 h before the remaining experimental visits. Participants completed their laboratory visits at the same time of day (± 2 h) to minimise the influence of circadian variations on the measurements. Participants were instructed to maintain their normal diet and physical activity for the duration of the study. Females were excluded from participating in the current study based on the potential for biological- and hormone-mediated heterogeneity to influence the study outcome measures. Indeed, it has been recently reported that menstrual cycle phase can influence skeletal muscle mitochondrial leak respiration and H_2_O_2_ production (Nelson et al. 2025).

### Experimental design

**Fig. 1:**
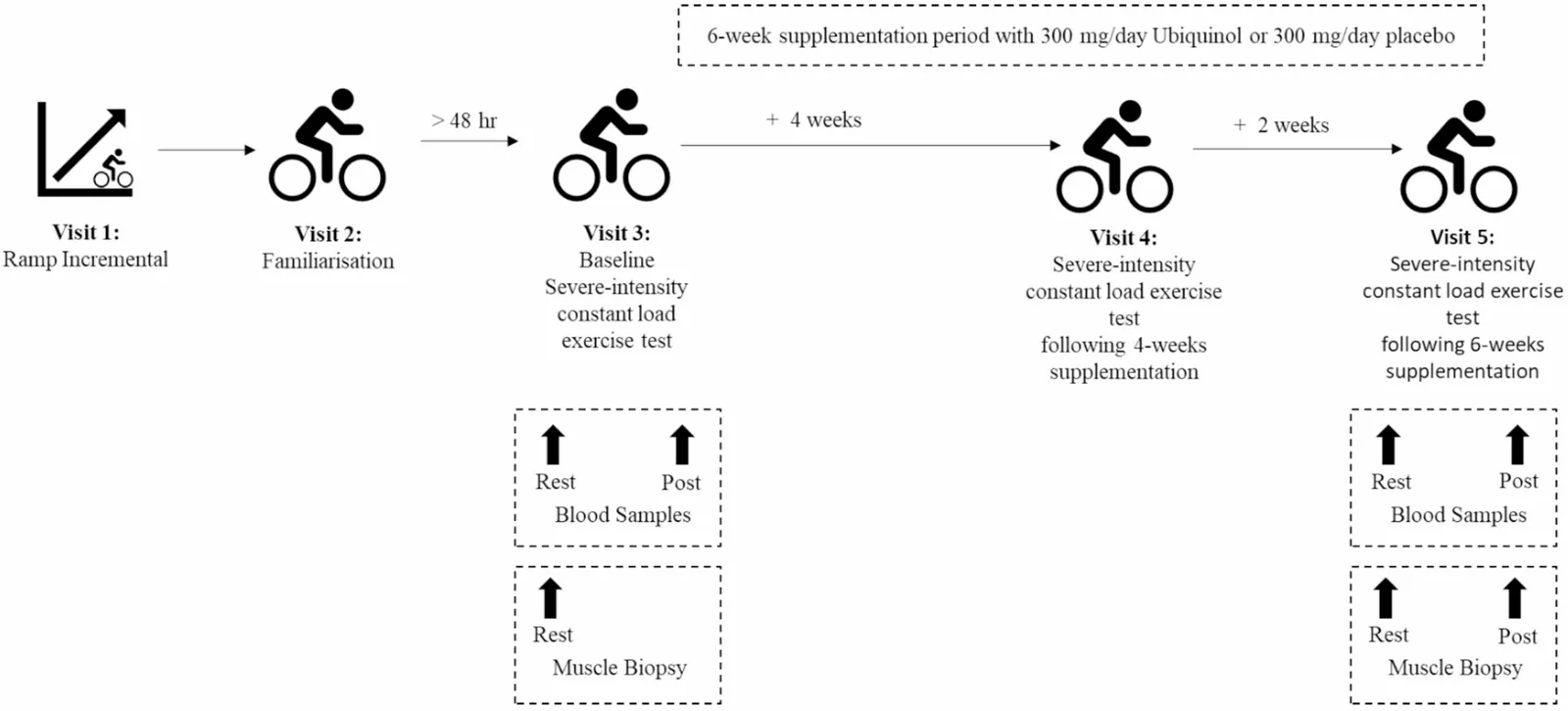
Schematic diagram outlining the study design

**Fig. 2:**
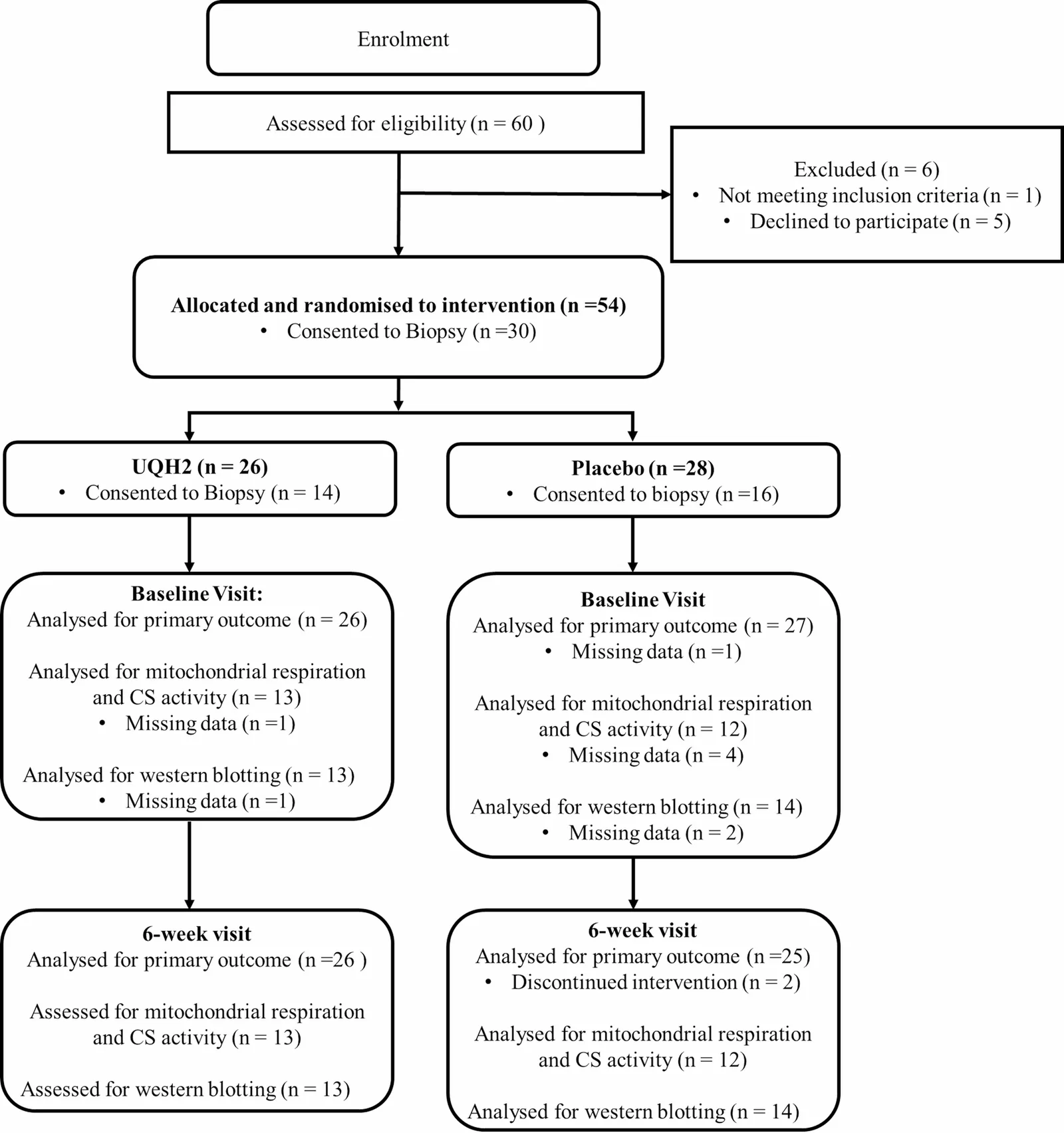
Flow diagram of participant progress through a randomised controlled trial with two experimental conditions, including information on enrolment, allocation to study, follow-up, and data analysis. The primary outcome was time to the limit of tolerance during a severe-intensity cycle test to exhaustion. Citrate synthase (CS)

Over a 7–9 week period, participants attended the laboratory on 5 separate occasions (Fig. 1). During the first visit to the laboratory, participants completed a ramp incremental exercise test to determine peak oxygen uptake (V̇O_2peak_) and gas exchange threshold (GET). On the second visit to the laboratory, participants were familiarised to the constant-load severe-intensity exhaustive exercise test. The third lab visit was the first experimental trial with baseline muscle biopsies obtained in participants who consented (*n* = 30) prior to a baseline constant-load cycling exercise test to exhaustion *(n* = 54). Subsequently, participants were stratified by V̇O_2peak_ and exercise performance, then randomly assigned to the ubiquinol (UQH_2_; mean ± SD: age, 24 ± 4 year; height, 1.77 ± 0.06 m; body mass, 76.7 ± 10.4 kg; V̇O_2peak_, 44.2 ± 4.8 mL∙kg^−1^∙min^−1^) or placebo (PLA; mean ± SD: age, 23 ± 5 year; height, 1.78 ± 0.07 m; body mass, 74.7 ± 8.6 kg; V̇O_2peak_, 46.7 ± 6.1 mL∙kg^− 1^∙min^−1^) supplementation groups, in a double-blind, independent groups experimental design (Fig. 2). Both participants and investigators were blinded to group assignment throughout the study, and placebo and ubiquinol capsules were identical in appearance, taste, odour and packaging. Allocation was concealed using opaque identical supplement containers, labelled A and B by an individual not involved in the study.

Participants were re-familiarised to the constant-load exercise tests after 4 weeks of a 6-week supplementation period (laboratory visit 4). A final constant-load exercise test was completed following a skeletal biopsy procedure (for the subsample who consented) after 6 weeks of supplementation, as described above for the third laboratory visit. All exercise tests were performed on an electronically braked cycle ergometer (Lode Excalibur Sport, Lode, Groningen, Netherlands). Preferred saddle and handlebar heights were recorded for each participant during visit 1 and replicated during subsequent visits.

### Incremental test

To determine V̇O_2peak_ and GET, participants initially completed a ramp incremental exercise test. Following 5 min baseline cycling at 50 W using a self-selected pedal cadence (70–90 rpm), work-rate increased by 30 W·min^−1^ until the limit of tolerance (T_Lim_). T_Lim_ was defined as the point of volitional exhaustion or when pedal cadence fell > 10 rpm below the self-selected pedal cadence for > 5 s. An online gas analyser (Vyntus CPX metabolic cart, Vyaire Medical, Chicago, USA) was used to measure breath-by-breath gas exchange and ventilation data during the test. Throughout the test, participants wore a face mask and breathed through a low-resistance volume transducer assembly with gas concentration signals continuously sampled via a capillary line connected to the volume transducer assembly. The gas analyser was calibrated with gases of known concentration, and the turbine volume transducer was calibrated automatically and manually with a 3 L syringe. Breath-by-breath pulmonary gas exchange and ventilation data were collected continuously during the incremental test and averaged over consecutive 10 s periods, with V̇O_2peak_ determined as the highest 30 s rolling mean value attained during the test. The GET was determined from the following three criteria: (1) the first disproportionate increase in CO_2_ production (V̇CO_2_) observed from individual plots of V̇CO_2_ vs. V̇O_2_; (2) an increase in the ventilatory equivalent for O_2_ (V̇_E_/V̇O_2_) without an increase in the ventilatory equivalent for CO_2_ (V̇_E_/V̇CO_2_); (3) an increase in end-tidal O_2_ tension without a drop in end-tidal CO_2_ tension. To account for the mean response time for V̇O_2_ during the ramp exercise protocol (i.e., the muscle-to-lung gas transit time) two-thirds of the ramp rate (20 W) was deducted from the work rates at GET and peak aerobic power (Whipp et al. 1981). Subsequently, the work rate at GET plus 70% of the difference in peak aerobic power and GET (∆) (70%∆) was calculated and administered in the subsequent severe-intensity step exercise tests.

### Experimental trials

After arriving at the laboratory, participants rested in a supine position for 10 min and a venous blood sample was collected. A skeletal muscle biopsy was subsequently collected prior to completion of a constant-load step incremental exercise test. For this test, participants completed 5 min of baseline cycling at 50 W, prior to an abrupt step increase in work rate to 70%∆. Verbal encouragement was provided during the test as participants were instructed to maintain their preferred pedal cadence (70–90 rpm) until T_Lim_, which was recorded using the same criteria outlined above for the ramp incremental test. T_Lim_ was recorded to the nearest second and was blinded to participants throughout the study. Upon completion, participants rested in a supine position for 10 min before a post-exercise venous blood sample was taken.

### Ubiquinol supplementation

Upon random assignment to the UQH_2_ or PLA group, participants consumed 300 mg·day^− 1^ of ubiquinol (Kaneka QH™, Kaneka corporation, Osaka, Japan) or placebo, respectively. This was spread evenly across the day in 100 mg doses consumed at breakfast, lunch, and dinner. Both the ubiquinol and placebo capsules contained edible oil, emulsifier, lecithin, modified food starch, carrageenan, caramel, and disodium phosphate encapsulated in a soft gel capsule. In addition to these ingredients, the ubiquinol and placebo capsules contained 100 mg ubiquinol or additional 100 mg edible oil, respectively. The purity of this ubiquinol supplement has been shown to be 96%, with ubiquinone and reduced coenzyme Q_10_ constituting the main impurities (Hosoe et al. 2007). Supplementation adherence was monitored by intentionally undersupplying participants with the required capsules to complete the study. Prior to depletion of their supply, participants were asked if any additional supplements were required to complete the study, and if so, how many.

## Ubiquinol supplementation

Upon random assignment to the UQH_2_ or PLA group, participants consumed 300 mg·day^− 1^ of ubiquinol (Kaneka QH™, Kaneka corporation, Osaka, Japan) or placebo, respectively. This was spread evenly across the day in 100 mg doses consumed at breakfast, lunch, and dinner. Both the ubiquinol and placebo capsules contained edible oil, emulsifier, lecithin, modified food starch, carrageenan, caramel, and disodium phosphate encapsulated in a soft gel capsule. In addition to these ingredients, the ubiquinol and placebo capsules contained 100 mg ubiquinol or additional 100 mg edible oil, respectively. The purity of this ubiquinol supplement has been shown to be 96%, with ubiquinone and reduced coenzyme Q_10_ constituting the main impurities (Hosoe et al. 2007). Supplementation adherence was monitored by intentionally undersupplying participants with the required capsules to complete the study. Prior to depletion of their supply, participants were asked if any additional supplements were required to complete the study, and if so, how many.

## Skeletal muscle analyses

### Muscle sampling and fibre preparation

Muscle biopsies were obtained from the vastus lateralis. Briefly, local anaesthetic was administered (1% lidocaine hydrochloride), a small incision (~ 1–2 cm) was then made through the skin and fascia using a scalpel, before a biopsy needle with suction was used to obtain the sample. The sample was blotted on blotting paper to remove excess blood and initially inspected to remove connective tissue and fat. Subsequently the sample was split into two portions. The first portion was immersed in ice-cold biopsy preservation solution (BioPS) for subsequent high-resolution respirometry analysis. The second portion was immediately snap frozen in liquid nitrogen for later analysis of citrate synthase (CS) activity, as a valid marker of mitochondrial content (Larsen et al. 2012), and for the determination of protein content of adenine nucleotide translocase 1 & 2 (ANT1 + 2), mitochondrial uncoupling protein 3 (UCP3) and oxidative phosphorylation (OXPHOS) complexes.

### Mitochondrial respiration protocol

Two 26-gauge needle tips were used to mechanically separate the muscle fibres under a light microscope, whilst immersed in BioPS. Upon separation, permeabilization of the plasma membrane was achieved by agitating the muscle fibre bundles for 30 min at 4 °C in BioPS containing 50 µg/ml saponin. The muscle fibre bundles were then washed in 2 mL of respiration medium (MIR05) containing, 0.5 mM EGTA,3 mM MgCl_2_, 60 mM potassium-lactobionate, 20 mM taurine, 10 mM KH_2_PO_4_, 20 mM 4-(2-hydroxyethyl)piperazine-1-ethanesulfonic acid (HEPES), 110 mM sucrose, and 1 g/L bovine serum albumin essentially fatty acid-free (pH 7.1). Muscle fibre bundles were removed from MIR05 and placed on filter paper for 2 s, then moved to a dry piece of filter paper for a further 2 s, weighed, and placed in fresh MIR05 (2 mL). Approximately 1–3 mg wet weight of muscle was added to 2 mL of MIR05 at 37 °C in the chamber of a high-resolution respirometer (Oxygraph-2k, Oroboros, Innsbruck, Austria) and mitochondrial respiration was assessed in quadruplicate. A hyper-oxygenated environment (200–500 µM O_2_) was maintained throughout the measurements with reoxygenation of the chamber achieved via direct injection of pure O_2_ to mitigate against oxygen diffusional limitation. The average coefficient of variation for measurement of CI + II_P_ respiration was 12.8%.

A substrate-uncoupler-inhibitor-titration protocol (SUIT 8) was used to determine leak respiration (_L_), ADP-stimulated respiration (_P_) and electron transfer system capacity (_E_) in mitochondrial protein complexes I – IV (CI – CIV). Steady states of O_2_ flux were marked on DatLab 7.3 (Oroboros, Innsbruck, Austria) following titrations in the following sequence: Pyruvate (5 mM) and Malate (2 mM) were added, in the absence of adenylates, enabling determination of leak respiration through CI (CI_L_). Subsequently, saturating concentrations of ADP (5 mM) were added to assess maximum ADP-stimulated respiration through CI (CI_P_). Cytochrome *c* (10 µM) was then added to assess the integrity of the outer mitochondrial membrane, with samples deemed to be compromised if respiration increased ≥ 15%; where this was violated, samples were removed from the final analysis. Glutamate (10 mM) was added to ensure maximal CI_p_ respiration was achieved. Maximal ADP-stimulated respiration through CI + II combined (CI + II_P_) was determined following the addition of succinate (10 mM). A series of stepwise titrations of carbonyl cyandide m-chlorophenyl hydrazone (0.5–1.5 µM) were added to uncouple respiration, enabling the assessment of ETS capacity through CI + II (CI + II_E_). Rotenone (0.5 µM) was added to inhibit CI and obtain ETS capacity through CII (CII_*E*_). Next, the addition of antimycin A (2.5 µM) inhibited complex III (CIII) allowing calculation of residual oxygen consumption, representing nonmitochondrial oxygen consumption, for the correction of mitochondrtial respiratory parameters. Artificial electron donors (Ascorbate, 2 mM) and N, N,N’,N’-tertamethyl-p-phenylenediamine (TMPD) (0.5 mM) were added to measure ETS through complex IV (CIV_*E*_). Finally, all mitochondrial respiration was inhibited and for the calculation of autoxidation of the O2k electrode facilitated following the addition of sodium azide (≥ 100 mM), allowing correction of CIV_E_. Respirometry values were averaged across the four experimental runs and expressed relative to muscle fibre wet weight and termed mass-specific mitochondrial respiration. Mass-specific respiration was also expressed relative to CS activity for correction to mitochondrial content (Larsen et al. 2012) and termed mitochondrial-specific respiration. Respiratory flux control ratios (FCR) were calculated and described in Eqs. 1–5.

1 $$\:\mathrm{L}\mathrm{C}\mathrm{R}=\frac{{\mathrm{C}\mathrm{I}}_{\mathrm{L}}}{\mathrm{C}\mathrm{I}+{\mathrm{C}\mathrm{I}\mathrm{I}}_{\mathrm{E}}}$$

2 $$\:\mathrm{P}\mathrm{C}\mathrm{R}=\:\frac{\mathrm{C}\mathrm{I}+{\mathrm{C}\mathrm{I}\mathrm{I}}_{\mathrm{P}}}{\mathrm{C}\mathrm{I}+{\mathrm{C}\mathrm{I}\mathrm{I}}_{\mathrm{E}}}$$

3 $$\:1/\mathrm{R}\mathrm{C}\mathrm{R}=\:\frac{{\mathrm{C}\mathrm{I}}_{\mathrm{L}}}{\mathrm{C}\mathrm{I}+{\mathrm{C}\mathrm{I}\mathrm{I}}_{\mathrm{P}}}$$

4 $$\:\mathrm{S}\mathrm{C}\mathrm{R}=\:\frac{{\mathrm{C}\mathrm{I}}_{\mathrm{P}}}{\mathrm{C}\mathrm{I}+{\mathrm{C}\mathrm{I}\mathrm{I}}_{\mathrm{P}}}$$

5 $$\:{\mathrm{C}\mathrm{I}\mathrm{V}}_{\mathrm{r}\mathrm{e}\mathrm{s}}=\frac{\mathrm{C}\mathrm{I}+{\mathrm{C}\mathrm{I}\mathrm{I}}_{\mathrm{P}}}{{\mathrm{C}\mathrm{I}\mathrm{V}}_{\mathrm{E}}}$$

Where LCR is the leak control ratio, PCR is the phosphorylation control ratio, inv-RCR is the inverse respiratory control ratio, SCR is the substrate control ratio and CIV_res_ is the CIV reserve ratio.

### Preparation of whole-muscle lysates

Approximately 10–20 mg of snap frozen muscle tissue was added to ice cold lysis buffer (1:10 w/v) containing PBS, 0.2% Triton X-100 and protease and phosphatase inhibitor cocktail (Fisher Scientific, Loughborough, UK). Samples were then blitzed twice for 2 min at 20 Hz using a tissue lyser (Tissue Lyser II, Qiagen, UK), rotated for 60 min at 4 °C and centrifuged at 1200 x*g* for 10 min. The resulting supernatant was frozen at -80 °C. The Pierce 660 protein assay (Fisher Scientific, Loughborough, UK) was used to determine, in triplicate, the protein concentration of the thawed muscle homogenate. The muscle homogenate was used for CS activity analysis.

### Citrate Synthase Activity

The following were added to a flat-bottomed 96-well plate to determine CS activity in triplicate: 40 µL of 3 mM acetyl CoA, 25 µL of 1 mM 5,5’-dithbiobis(2-nitrobenzoic acid) (DTNB) solution, 160 µL of 100 mM Tris buffer containing 1% Triton-X (pH 8.3) and 10 µL of a 1 mg∙mL^− 1^ muscle homogenate. Immediately after the addition of 15 µL of 10 mM oxaloacetate to all wells, the plate was shaken for 30 s and read every 30 s for 6 min at 412 nm at 30 °C using a microplate reader (Varioskan Lux, ThermoFisher, Loughborough, UK). Absorbance values were corrected to blank wells and pathlength corrections applied. CS activity was determined over a 3 min period following exclusion of the initial min of the reaction and reported as mol∙h^− 1^∙kg protein^− 1^.

### Immunoblot analysis

A portion of homogenate was then mixed with 4x Laemmli sample buffer (Bio-rad, Herts, UK), 0.1% β-mercaptoethanol (Sigma, Dorset, UK) and deionised water to the required concentration. Samples were then boiled for 5 min at 95 °C for ANT1 + 2, and UCP3 but not Total OXPHOS. The appropriate sample load was determined prior to analysis for each antibody (Total OXPHOS, 20 µg; ANT1 + 2, 10 µg; UCP3, 10 µg) and loaded on to 4–20% (Total OXPHOS) or 4–15% (UCP3 & ANT1 + 2,) stain-free TGX polyacrylamide gels (Bio-rad, Herts, UK) and separated by gel electrophoresis at 150 V for ~ 1 h. Protein was transferred to PVDF membranes using a Trans-Blot turbo system (Bio-rad, Herts, UK). Membranes underwent 3 × 5 min washes in Tris-buffered saline with tween before being blocked in EveryBlot blocking buffer (Bio-rad, Herts, UK) for 5 min and then washed again as before. Membranes were incubated overnight at 4 °C with the primary antibodies: Total OXPHOS 1:2000 (AB110411, abcam, Cambridge, UK), ANT1-2 1:1000 (AB220408, abcam, Cambridge, UK) and UCP3 1:1000 (AB3046, abcam, Cambridge, UK). Primary antibodies were diluted in EveryBlot blocking buffer. Membranes were washed as before and incubated with anti-mouse (Total OXPHOS; 7076, Cell Signalling Technology, London UK) or anti-rabbit (ANT1 + 2 & UCP3; 7074, Cell Signalling Technology, London UK) horseradish peroxidase-conjugated secondary antibodies for 1 h at room temperature. Secondary antibodies were diluted 1:10000, in Everyblot blocking buffer. Membranes were washed as before and incubated with enhanced chemiluminescence substrate (ClarityMax, Bio-Rad, Herts, UK) for 5 min. Visualisation of the membranes was achieved using a ChemiDocTM imaging system (Bio-rad, Herts, UK). Image analysis software (Image Lab v. 4.0, Bio-Rad, Herts, UK) was used for densiometric analysis of protein bands with the target signal expressed relative to total lane protein on a stain free gel.

## Venous blood analyses

### Venous blood sampling and treatment

Venous blood was collected into lithium-heparin and serum separator vacutainers. Lithium-heparin vacutainers were immediately spun at 1620 *g* and 4 °C for 10 min. Serum separator vacutainers were left at room temperature for 30 min prior to centrifugation at 1620 *g* and 4 °C for 10 min to obtain serum.

### Plasma coenzyme Q_10_ content

Lithium-heparin plasma (100 µL) was added to deoxygenated 1-propanol (300 µL) and butylated hydroxytoluene (40 µL; 10 mg/mL in ethanol) and then vortexed for 10 s before being snap frozen in liquid nitrogen. Kaneka Techno Research Corporation (Osaka, Japan) analysed the concentration of plasma UQ and UQH_2_ using liquid chromatography (LC)-mass spectrometry (MS)/MS method as described previously (Suzuki et al. 2021). Kaneka Research Corporation were blinded to the experimental conditions and this analysis was undertaken once all other analysis for the study had been completed.

### Serum lipid hydroperoxides

Serum lipid hydroperoxides (LOOH) were measured spectrophotometrically using a modification of the Wolff et al. (1994) method as described previously (Williamson et al. 2020). The ferrous oxidation in xylenol orange (FOX-1) reagent containing ammonium ferrous sulphate (250 µM), sorbitol (100 mM), xylenol orange (100 µM), and sulphuric acid (25 mM) was used. Briefly, 0–5.0 µmol∙L^− 1^ of hydrogen peroxide was used to form a standard curve. Serum (90 µL) was added to FOX-1 reagent (900 µL). After a 30 min incubation in the dark, 200 µL of this solution was added to a 96 well plate, and immediately read using a microplate reader (Varioskan Lux, ThermoFisher, Loughborough, UK) at 560 nm and then every 2 min for 6 min to confirm the reaction rate had saturated.

### Blood lipid profile

Serum triglycerides, high-density lipoproteins (HDL), total cholesterol were analysed spectrophotometrically using a semi-automatic analyser (Pentra C400, Horiba Medical, Northampton, UK). The Friedewald equation was used to estimate low-density lipoprotein cholesterol (LDL) (Friedewald et al. 1972).

### Measurements

Breath-by-breath pulmonary gas exchange and ventilation were measured continuously using an online gas analyser (Vyntus CPX; Carefusion, California, USA) as described above for the ramp incremental test. Errant breaths falling more than 4 standard deviations outside the local mean were removed during initial examination of the data. The magnitude of the V̇O_2_ slow component (V̇O_2_ SC) was inferred from the difference in V̇O_2_ between 120 ± 10 s and T_lim_ (averaged over final 30 s).

### Statistical analysis

Sample size was estimated with a priori power analysis using G*Power (G*Power 3.1.97; Kiel, Germany). Since the effects of UQH_2_ supplementation on our primary outcome (T_Lim_ during severe-intensity cycling exercise) had not been assessed previously, sample size to detect a supplement x time interaction effect for two supplements (UQH_2_ vs. PLA) and two time points (Pre & Post-supplementation) was estimated on the basis of a small effect size (0.20), a statistical power of 0.80, a significance level of 0.05. Inputting these values into G*Power revealed that a sample size of 52 was required. Statistical analysis was conducted using jamovi (version 2.3.28; Jamovi, Sydney, Australia). Data were tested for normality by visual inspection of Q-Q plots of residuals, residuals histogram and Shapiro-Wilk tests. Linear mixed models (2 [supplement: UQH_2_ vs. PLA] x 2 [time: Pre & Post-supplementation]) were performed to determine differences in T_Lim_, plasma CoQ_10_ content variables, mitochondrial respiratory function variables, CS activity, LOOH and serum blood lipid variables. Effect sizes were calculated using partial eta squared (*n*_*p*_^*2*^) for linear mixed models outcomes and interpreted as small (0.01–0.05), moderate (0.06–0.13), and large (≥ 0.14). Where significant supplement × time interaction effects were observed, Holm-Bonferroni corrected independent *t*-tests (between group comparisons) and paired *t*-tests (within group comparisons) were used for *post-hoc* analysis. Independent samples *t*-tests were used to determine differences between UQH_2_ and PLA in fold change in protein content of ANT1 + 2, UCP3 and OXPHOS complexes. Effect sizes were calculated and reported for all *t*-tests using Hedge’s *g* bias correction and interpreted as ‘small’ (0.20–0.49), ‘moderate’ (0.50–0.79), and large (≥ 0.80). All data are presented as mean ± SD. Significance was accepted at *P* ≤ 0.05.

## Results

All participants requested additional capsules in line with the amount undersupplied, self-reported that they consumed all supplements as instructed, and returned empty capsule pots. No adverse side effects were reported in either the UQH_2_ or PLA supplementation conditions.

### Plasma CoQ_10_ profile

**Fig. 3:**
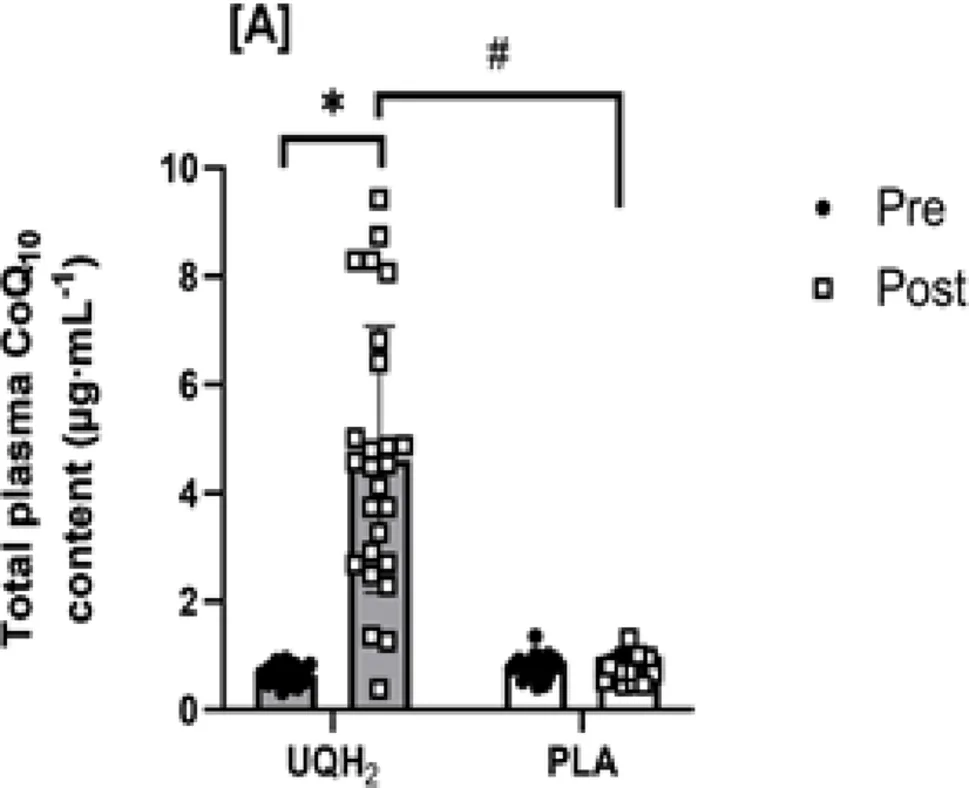
Total Coenzyme Q_10_ (CoQ_10_) content in plasma pre and post supplementation with ubiquinol (UQH_2_; *n* = 26 at both timepoints) or placebo (PLA; *n* = 24 at Pre and *n* = 22 at Post). *indicates a statistically significant change (*P* < 0.05) pre to post within groups ^#^ indicates a statistically significant change (*P* < 0.0001) at Post between groups. Statistical significance was accepted as *P* ≤ 0.05

There was a supplement × time interaction effect (*P* < 0.0001; *n*_*p*_^*2*^ ≥ 0.17 ≤ 0.58) and main effects of supplement (*P* ≤ 0.002; *n*_*p*_^*2*^ ≥ 0.17 ≤ 0.52) and time (*P* < 0.0001; *n*_*p*_^*2*^ ≥ 0.43 ≤ 0.58) for plasma total CoQ_10_ (Fig. 3), UQH_2_, UQ, total CoQ_10_/Cholesterol and total CoQ_10_/LDL. These measures were significantly higher Post compared to Pre following UQH_2_ (*P* < 0.0001, *g* > 2.0) but did not change following PLA supplementation (*P* > 0.05). In addition, these measures were higher at Post in UQH_2_ compared to PLA supplementation (*P* < 0.0001, *g* > 2.0). There was no supplement × time interaction effect (*P* = 0.37; *n*_*p*_^*2*^ = 0.02) or no main effect of supplement (*P* = 0.34; *n*_*p*_^*2*^ = 0.02), but a main effect for time (*P* = 0.04; *n*_*p*_^*2*^ = 0.08), for UQH_2_/Total CoQ_10_ (Table 1).

### Serum lipid profile

There was no supplement × time interaction effect (*P* ≥ 0.51 ≤ 0.79; *n*_*p*_^*2*^ < 0.01), or no main effect of time (*P* ≥ 0.07 ≤ 0.95; *n*_*p*_^*2*^ < 0.01) or supplement (*P* ≥ 0.55 ≤ 0.81; *n*_*p*_^*2*^ < 0.01), for total cholesterol, triglycerides, LDL, and HDL concentration (Table 1).

**Table 1:** Plasma Coenzyme Q_10_ and serum lipid profiles pre and post supplementation with ubiquinol or Placebo

	Ubiquinol			Placebo		
	Pre	Post	Mean diff	Pre	Post	Mean diff
*Coenzyme Q10 profile*						
Total CoQ_10_ (µg∙mL^− 1^)	0.65 ± 0.16	4.62 ± 2.46* ^**#**^	3.97 ± 2.38	0.79 ± 0.20	0.76 ± 0.20	-0.01 ± 0.17
Ubiquinol (µg∙mL^− 1^)	0.59 ± 0.15	4.35 ± 2.35* ^**#**^	3.76 ± 2.29	0.69 ± 0.18	0.68 ± 0.21	0.01 ± 0.17
Ubiquinone (µg∙mL^− 1^)	0.06 ± 0.04	0.27 ± 0.17* ^**#**^	0.21 ± 0.14	0.10 ± 0.08	0.08 ± 0.06	-0.02 ± 0.06
Ubiquinol/Total CoQ_10_ (%)	89.8 ± 6.1	92.6 ± 5.2	2.74 ± 3.44	88.8 ± 7.3	90.0 ± 10.3	1.05 ± 8.59
Total CoQ_10_/Cholesterol	0.17 ± 0.04	1.16 ± 0.60* ^**#**^	0.99 ± 0.58	0.20 ± 0.04	0.20 ± 0.06	0.01 ± 0.05
Total CoQ_10_/LDL	0.31 ± 0.10	2.21 ± 1.14* ^**#**^	1.89 ± 1.11	0.37 ± 0.12	0.39 ± 0.23	0.02 ± 0.22
*Serum lipid profile*						
Total Cholesterol (mmol∙L^− 1^)	3.92 ± 0.87	3.96 ± 0.67	0.04 ± 0.45	4.05 ± 0.83	4.02 ± 0.75	-0.03 ± 0.47
Triglycerides (mmol∙L^− 1^)	0.94 ± 0.35	1.10 ± 0.54	0.16 ± 0.47	1.00 ± 0.36	1.09 ± 0.64	0.08 ± 0.43
LDL (mmol∙L^− 1^)	2.21 ± 0.68	2.14 ± 0.55	-0.07 ± 0.36	2.25 ± 0.77	2.18 ± 0.73	-0.07 ± 0.39
HDL (mmol∙L^− 1^)	1.29 ± 0.30	1.34 ± 0.27	0.04 ± 0.13	1.34 ± 0.24	1.34 ± 0.29	0.01 ± 0.22

Values are Mean ± SD. Total CoQ_10_, total coenzyme Q_10_

*LDL* low-density lipoprotein; *HDL*, high density lipoprotein

*n* = 26 in all measures in the ubiquinol group at both timepoints. In the placebo group *n* = 24 at Pre and *n =* 23 at Post for Total CoQ_10_, Ubiquinol, Ubiquinone, and Ubiquinol/Total CoQ_10_ measures and *n* = 24 at Pre and *n* = 22 at Post and *n* = 27 at Pre and *n* = 24 at Post for serum lipid profile measures

*Indicates statistically significant difference from PRE value,

^#^ indicates statistically significant difference between groups at POST time point. Statistical significance was accepted as *P* ≤ 0.05

### Citrate synthase activity

**Fig. 4:**
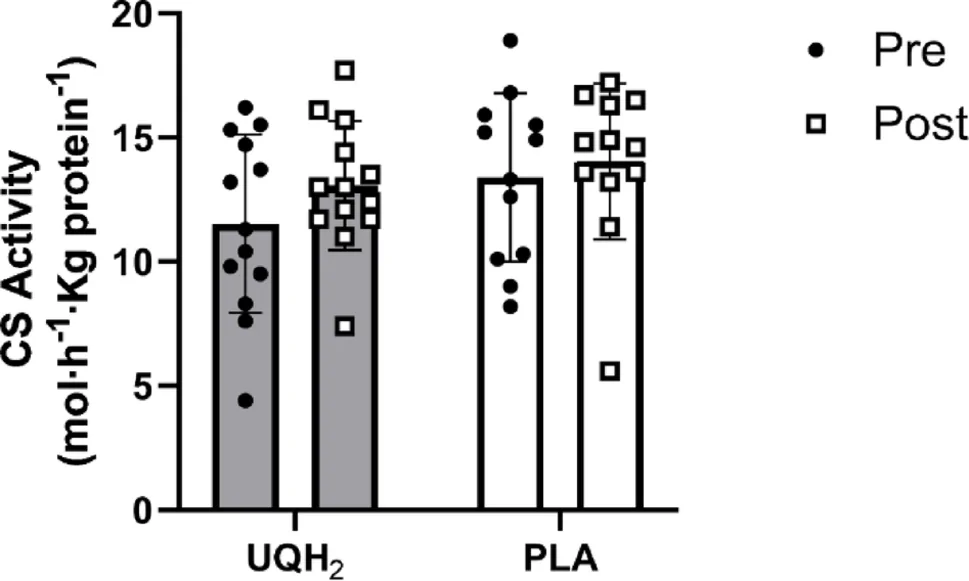
Citrate synthase (CS) activity as a biomarker of mitochondrial content pre and post ubiquinol (UQH_2_; *n* = 13 at both time points) or placebo (PLA; *n* = 12 at both time points) supplementation

There was no supplement x time interaction effect (*P* = 0.51; *n*_*p*_^*2*^ = 0.02), or main effects of time (*P* = 0.11; *n*_*p*_^*2*^ = 0.11) or supplement (*P* = 0.21; *n*_*p*_^*2*^ = 0.07), for CS activity (Fig. 4).

### Mitochondrial respiratory function

**Fig. 5:**
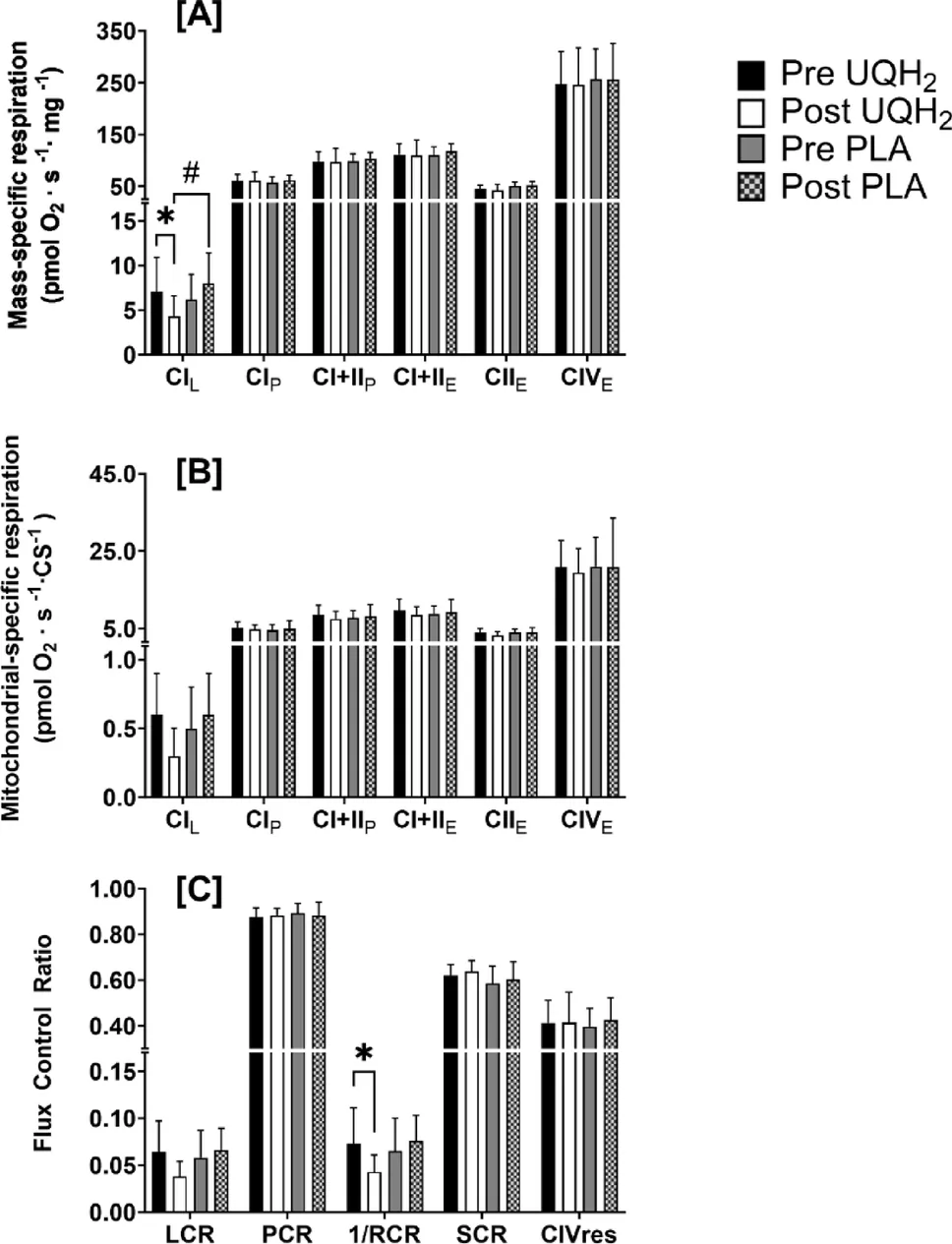
Mitochondrial mass-specific respiration (**A**), mitochondrial-specific respiration (**B**) and flux control ratios (FCRs) (**C**) pre (Pre UQH_2_) and post (Post UQH_2_) ubiquinol supplementation and pre (Pre PLA) and post (Post PLA) placebo supplementation. UQH_2_; *n* = 13 at both time points for panel A-C, PLA; *n* = 12 at both timepoints for panels A-C. *Indicates statistically significant change from Pre to Post supplementation within groups and ^#^ indicates statistically significant difference Post supplementation between groups. Statistical significance was accepted as *P* ≤ 0.05

There was a supplement × time interaction effect (*P* = 0.004; *n*_*p*_^*2*^ = 0.32), but no main effect of time (*P* = 0.48; *n*_*p*_^*2*^ = 0.02) or supplement (*P* = 0.20; *n*_*p*_^*2*^ = 0.07), for mass-specific CI_L_ respiration. Post-hoc tests revealed that CI_L_ respiration was lower in the UQH_2_ group (4.3 ± 2.3 pmol O_2_∙s^− 1^∙mg^− 1^) compared to the PLA group (8.0 ± 3.4 pmol O_2_∙s^− 1^∙mg^− 1^) POST supplementation (*P* = 0.024, *g* = 0.87). CI_L_ was lower Post compared to Pre (7.1 ± 3.8 pmol O_2_∙s^− 1^∙mg^− 1^) in the UQH_2_ group (*P* = 0.016, *g* = 0.85) (Fig. 5A). There was a supplement × time interaction effect (*P* = 0.011; *n*_*p*_^*2*^ = 0.27), but no main effects of time (*P* = 0.364; *n*_*p*_^*2*^ = 0.04) or supplement (*P* = 0.247; *n*_*p*_^*2*^ = 0.06), for mitochondrial-specific CI_L_ respiration. Post-hoc tests revealed that mitochondrial-specific CI_L_ respiration was lower Post (0.34 ± 0.19 pmol O_2_∙s^− 1^∙mg^− 1^/CS activity) compared to Pre (0.57 ± 0.33 pmol O_2_∙s^− 1^∙mg^− 1^/CS activity) in the UQH_2_ group (*P* = 0.026, *g* = 0.80) but was not different in the UQH_2_ group compared to the PLA (0.62 ± 0.34 pmol O_2_∙s^− 1^∙mg^− 1^/CS activity) group POST supplementation (*P* = 0.08, *g* = 0.69) (Fig. 5B).

There was no supplement × time interaction effect (*P* ≥ 0.18 ≤ 0.99; *n*_*p*_^*2*^ ≤ 0.08), or no time (*P* ≥ 0.34 ≤ 0.94; *n*_*p*_^*2*^ ≤ 0.07) and supplement (*P* ≥ 0.59 ≤ 0.99; *n*_*p*_^*2*^ ≤ 0.01) main effects, for mass-specific or mitochondrial-specific CI_P_, CI + II_P_, CI + II_E_ or CIV_E_ respiration (Fig. 5). There was no supplement x time interaction effect (*P* = 0.21; *n*_*p*_^*2*^ = 0.07) or no main effect for time (*P* = 0.66; *n*_*p*_^*2*^ < 0.01), but a main effect for supplement (*P* = 0.03; *n*_*p*_^*2*^ = 0.20), for mass-specific CII_E_ respiration (Fig. 4A). However, there was no supplement x time interaction effect (*P* = 0.25; *n*_*p*_^*2*^ < 0.06), or no main effect of time (*P* = 0.22; *n*_*p*_^*2*^ = 0.07) or supplement (*P* = 0.27; *n*_*p*_^*2*^ = 0.05), for mitochondrial specific CII_E_ respiration (Fig. 5B).

### Flux control ratios

There was a supplement x time interaction effect (*P* = 0.02; *n*_*p*_^*2*^ = 0.22) for 1/RCR, but no main effects of time (*P* = 0.15; *n*_*p*_^*2*^ = 0.09) or supplement (*P* = 0.348; *n*_*p*_^*2*^ = 0.04). Post-hoc tests revealed 1/RCR was lower at Post (0.044 ± 0.019) compared to Pre (0.073 ± 0.039) supplementation in the UQH_2_ group (*P* = 0.014, *g* = 0.88) (Fig. 5C), but was not significantly different Post supplementation in the UQH_2_ group compared to the PLA group (0.072 ± 0.026, *P* = 0.16, *g* = 1.20). There was a supplement x time interaction effect (*P* = 0.02; *n*_*p*_^*2*^ = 0.22), but no main effects of time (*P* = 0.18; *n*_*p*_^*2*^ = 0.08) or supplement (*P* = 0.27; *n*_*p*_^*2*^ = 0.06), for LCR. Post-hoc tests revealed LCR was lower POST (0.038 ± 0.016) compared to PRE (0.063 ± 0.034) in the UQH_2_ group (*P* = 0.02, *g* = 0.88) (Fig. 5C).

There were no supplement x time interaction effects (*P* ≥ 0.49 ≤ 0.64; *n*_*p*_^*2*^ ≤ 0.02), or no main effects for time (*P* ≥ 0.29 ≤ 0.71; *n*_*p*_^*2*^ ≤ 0.05) or supplement (*P* ≥ 0.08 ≤ 0.32; *n*_*p*_^*2*^ ≤ 0.13), for PCR, SCR, and CIV_Res_ (Fig. 5C).

### Immunoblot analysis

**Fig. 6:**
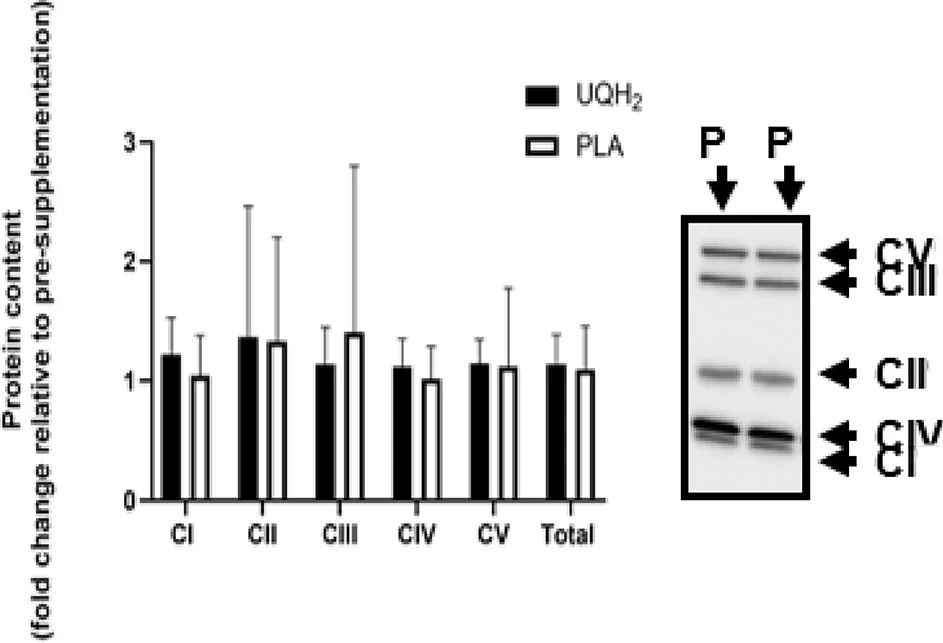
Fold change compared to pre-supplementation of the protein content of mitochondrial complex I (CI), II (CII), III (CIII), IV (CIV), V (CV) and total mitochondrial OXPHOS complex content (Total) following ubiquinol (UQH_2_; *n* = 13) supplementation and placebo (PLA; *n* = 14). A representative blot from one participant is shown on the right

**Fig. 7:**
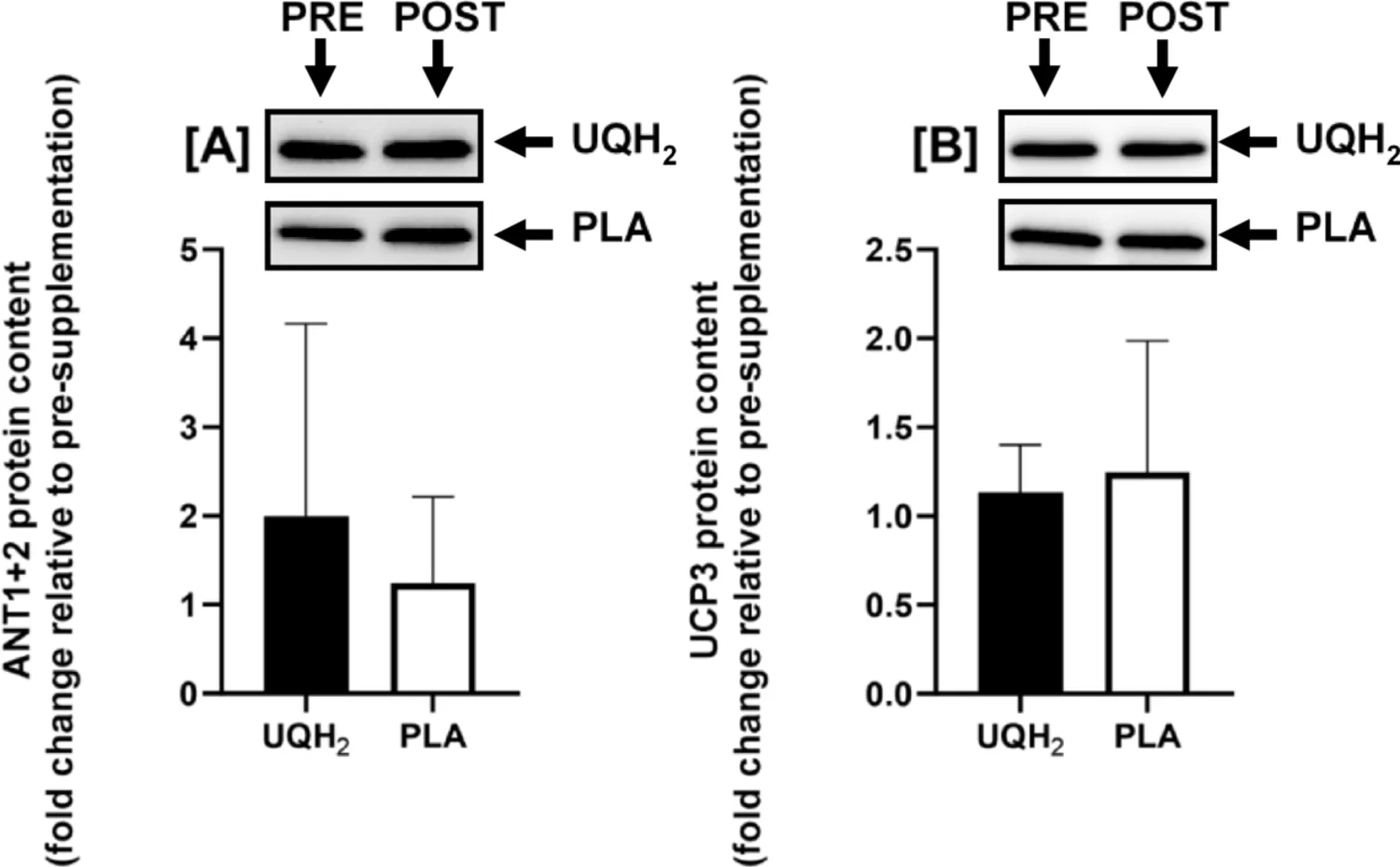
Fold change compared to pre-supplementation of the protein content of adenine nucleotide translocase 1 + 2 (ANT1 + 2; A) and mitochondrial uncoupling protein 3 (UCP3; B) following ubiquinol (UQH_2_; *n* = 13) supplementation and placebo (PLA; *n* = 14). Representative blots from one participant are shown for each protein

There were no differences in fold change in protein content from PRE to POST supplementation between groups in any of the individual OXPHOS complexes (*P* ≥ 0.171, *g* = 0.05–0.53; Fig. 6), ANT1 + 2 (*P* = 0.247, *g* = 0.44; Fig. 7A) or UCP3 (*P* = 0.609, *g* = 0.19; Fig. 7B).

### Performance measures

#### T_Lim_

**Fig. 8:**
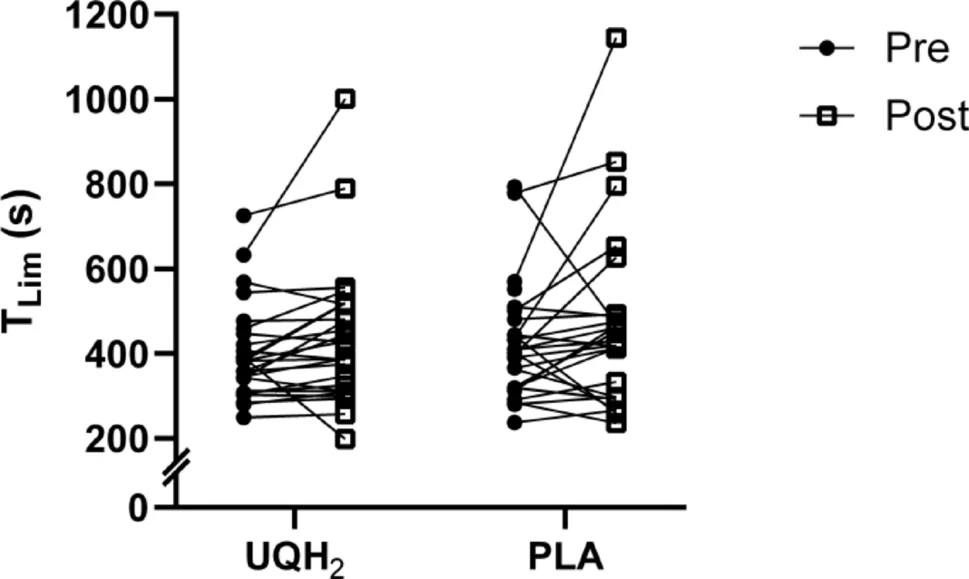
Time to reach the limit of tolerance (T_Lim_) during the severe intensity constant-load cycling test pre and post 6-weeks supplementation with ubiquinol (UQH_2_; *n* = 26 at both timepoints) or placebo (PLA; Pre, *n* = 27, Post, *n* = 25)

There was no supplement x time interaction effect (*P* = 0.79; *n*_*p*_^*2*^ < 0.01) or no main effect of effect supplement (*P* = 0.44; *n*_*p*_^*2*^ = 0.01), but there was a main effect of time (*P* = 0.03; *n*_*p*_^*2*^ = 0.08), for T_Lim_ (Fig. 8).

### Pulmonary oxygen uptake

**Fig. 9:**
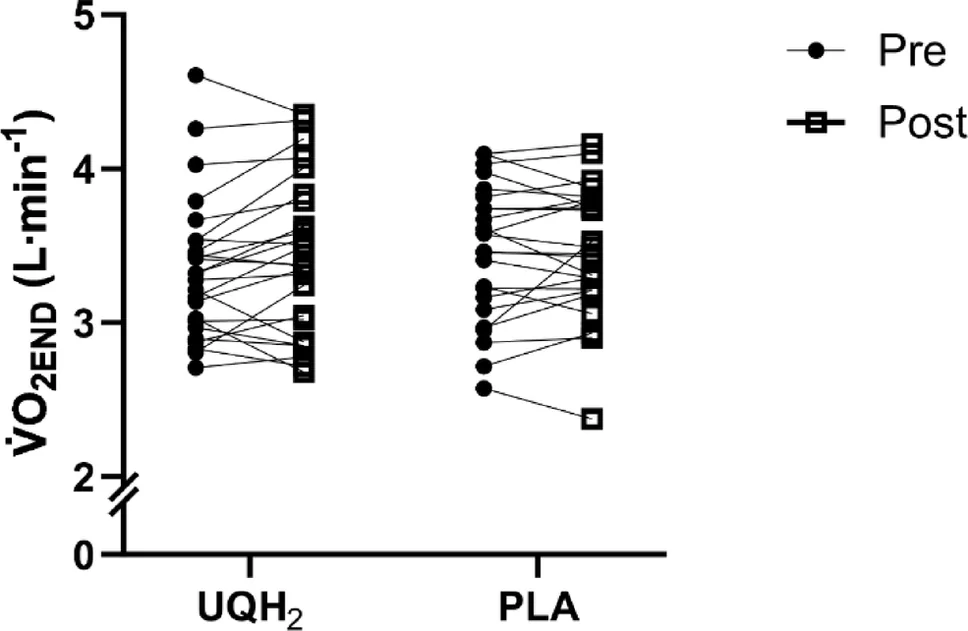
Oxygen uptake averaged over the final 30 s of the constant load exercise test (V̇O_2END_) Pre and Post 6-weeks supplementation with ubiquinol (UQH_2_; *n* = 25 at both timepoints) or placebo (PLA; Pre, *n* = 26, Post, *n* = 24)

There was no supplement x time interaction effect (*P* = 0.25; *n*_*p*_^*2*^ = 0.03), or no main effects of time (*P* = 0.11; *n*_*p*_^*2*^ = 0.05) or supplement (*P* = 0.34; *n*_*p*_^*2*^ = 0.02), for end-exercise V̇O_2_ averaged over the final 30s of the constant-load exercise tests (Fig. 9).

**Fig. 10:**
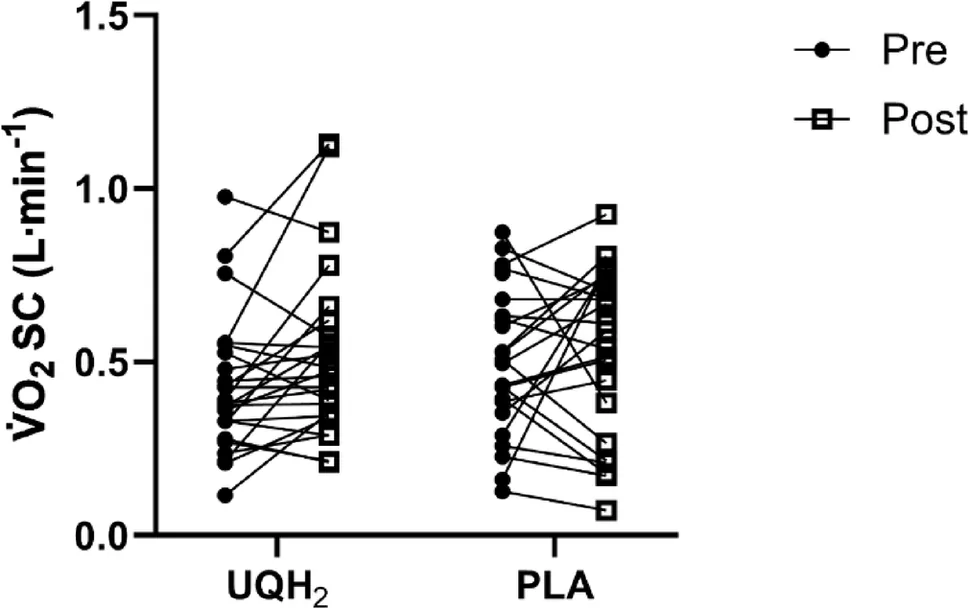
Oxygen uptake (V̇O_2_) slow component (V̇O_2_SC) assessed as the difference in V̇O_2_ between V̇O_2_ averaged over the final 30 s of the constant load test (V̇O_2END_) and at 120 ± 15 s Pre and Post 6-weeks supplementation with ubiquinol (UQH_2_; *n* = 25 at both timepoints) or placebo (PLA; Pre, *n* = 26, Post, *n* = 24)

There was no supplement x time interaction effect (*P* = 0.38; *n*_*p*_^*2*^ = 0.02) or main effect of supplement (*P* = 0.53; *n*_*p*_^*2*^ < 0.01), but there was a main effect of time (*P* = 0.01; *n*_*p*_^*2*^ = 0.12), for V̇O_2_ SC (Fig. 10).

### Serum lipid hydroperoxides (LOOH)

**Fig. 11:**
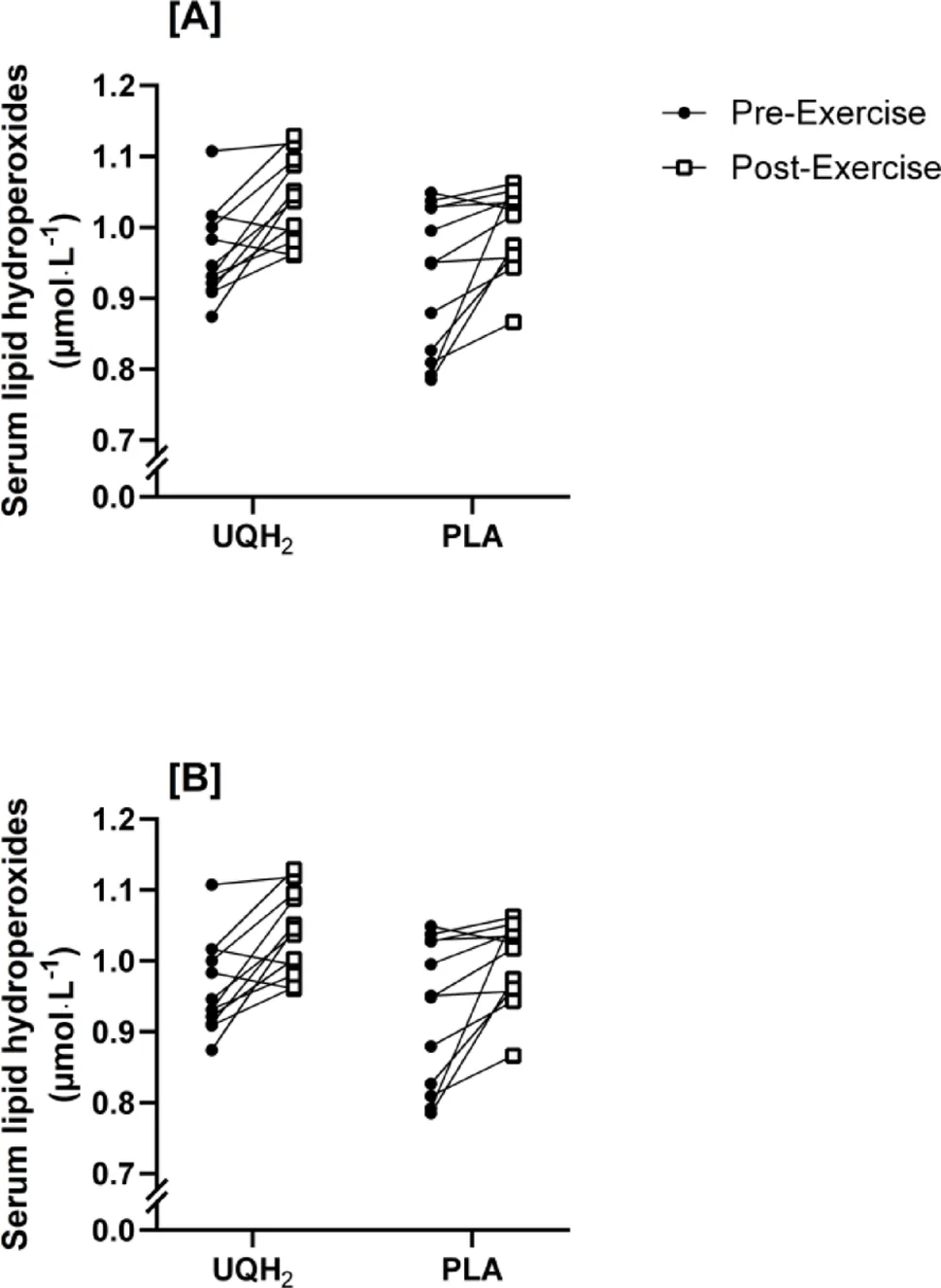
Pre- and post-exercise serum lipid hydroperoxides both pre (A) and post supplementation (B), in the ubiquinol (UQH_2_; *n* = 12 at both timepoints) and placebo (PLA; *n* = 12 at both timepoints) group

There was no supplement x time interaction effect (*P* ≥ 0.37 ≤ 0.89; *n*_*p*_^*2*^ ≤ 0.04) or no main effect for supplement (*P* ≥ 0.17 ≤ 0.58; *n*_*p*_^*2*^ ≥ 0.01 ≤ 0.08), but there was a main effect for time (*P* < 0.004; *n*_*p*_^*2*^ = ≥ 0.32 ≤ 0.50), for LOOH both pre and post supplementation (Fig. 11).

## Discussion

The principal original finding of the current study, consistent with our experimental hypothesis, was that 6 weeks UQH_2_ lowered CIL respiration and 1/RCR suggesting improved mitochondrial oxidative phosphorylation coupling efficiency. However, contrary to our experimental hypothesis, this improved mitochondrial respiratory coupling efficiency was not mediated by lower skeletal muscle ANT1 + 2 and UCP-3 protein content and did not translate to improved T_Lim_ during severe-intensity exercise. These findings indicate that UQH_2_ supplementation was effective at improving mitochondrial respiratory coupling efficiency but not enhancing severe-intensity exercise T_Lim_.

Consistent with previous studies, UQH_2_ supplementation increased plasma [CoQ10] (Langsjoen and Langsjoen 2014; Zhang et al. 2018). The principal original findings from this study were that 6 weeks supplementation with 300 mg/day UQH_2_ lowered 1/RCR and LCR. Lower 1/RCR and LCR indicate that leak respiration compromised a smaller fraction of maximal ADP-stimulated respiration and maximal ETS activity after UQH_2_ supplementation, with lower 1/RCR suggesting that the efficiency of mitochondrial oxidative phosphorylation was improved. These findings were underpinned by lower CI_L_ respiration, concomitant with no change in CI_P_, CI + II_P_, CI + II_E_ nor CII_E_ after UQH_2_ supplementation. In the only other study to have assessed the effect of UQH_2_ supplementation on mitochondrial respiratory variables in human skeletal muscle, Pham et al. (2020) reported that 6 weeks supplementation with 200 mg/day UQH_2_ did not alter CI_L_, CI + II_L_, CI + II_P_ nor CI + II_E_. It is possible, therefore, that the higher daily dose of UQH_2_ administered in the current study compared with Pham et al. (2020) accounted for the between-study difference in CI_L_. However, both the current study and the study of Pham et al. (2020) did not observe improvements in CI + II_P_ or CI + II_E_ respiratory rates, or muscle CS activity, nor the protein content of mitochondrial respiratory complexes I-V as markers of mitochondrial content.

The lowering in CI_L_ respiration after UQH_2_ supplementation occurred independent of altered skeletal muscle protein content of the mitochondrial leak proteins, ANT1 + 2 and UCP-3. It is possible that the lower CI_L_ respiration in the current study was linked to lower ETS electron leak as ROS, and therefore improved efficiency of mitochondrial electron handling, as previously reported after UQH_2_ supplementation (Pham et al. 2020). Lower ROS formation after UQH_2_ supplementation could also lower leak respiration by mitigating oxidative damage, via lipid peroxidation, to the mitochondrial membranes (Nicholls and Ferguson 2013) with ROS and lipid peroxidation derivatives also able to acutely increase ANT1 + 2 and UCP-3 activity (Echtay et al. 2003; Klingenberg and Winkler 1985). Although circulating lipid hydroperoxides, as systemic markers of lipid peroxidation (Fogarty et al. 2011), were not altered, it cannot be excluded that UQH_2_ supplementation attenuated lipid peroxidation in the muscle mitochondria, or attenuated muscle mitochondrial ROS production, in the current study. Indeed, the previous observation of lower mitochondrial H_2_O_2_ emission per unit respiration during leak respiration after UQH_2_ supplementation occurred independent of changes in plasma TBARS and urinary isoprostanes as systemic markers of lipid peroxidation (Pham et al. 2020). Another candidate mechanism for the lower CI_L_ respiration after UQH_2_ supplementation in the current study is lower electron slip, whereby electrons are transferred along the mitochondrial ETS in the absence of H^+^ translocation into the mitochondrial intermembrane space (Fang et al. 2020; Jastroch et al. 2010).

Despite enhanced mitochondrial oxidative phosphorylation efficiency, UQH_2_ supplementation did not increase severe-intensity exercise T_Lim_. The lack of an ergogenic effect of UQH_2_ supplementation in the current study is consistent with some (Bloomer et al. 2012; Orlando et al. 2018), but not all (Alf et al. 2013; Moreno-Fernandez et al. 2023), previous studies. Moreover, and in keeping with the findings of the current study, some previous studies have also dissociated improved mitochondrial oxidative phosphorylation efficiency from exercise performance enhancements(Ghiarone et al. 2019; Jacobs et al. 2012; Peden et al. 2022). Whist it is not possible to exclude that a longer duration of supplementation and/or a higher UQH_2_ dose could have elicited an ergogenic effect, the UQH_2_ supplementation regime in the current study has been previously reported to enhance endurance performance (Alf et al. 2013). In addition to a lack of effect on severe-intensity exercise T_Lim_, UQH_2_ supplementation did not alter the change in V̇O_2_ between 2 min and end-exercise, as a marker of exercise economy, or end-exercise V̇O_2_ which is equivalent to V̇O_2max_ during severe-intensity exercise (Burnley and Jones 2007). Previous studies have also dissociated changes in mitochondrial oxidative phosphorylation efficiency from exercise economy and V̇O_2max_ enhancements (Ghiarone et al. 2019; Jacobs et al. 2012). Given that 200 mg/day UQH_2_ supplementation for 2 weeks improved resistance exercise performance (Moreno-Fernandez et al. 2023), whereas 300 mg/day UQH_2_ supplementation for 6 weeks was required to improve endurance performance (Alf et al. 2013), it is possible that UQH_2_ supplementation may hold greater ergogenic potential for resistance exercise performance and recovery, but this requires further research as the conclusions from these studies may have been based on a Type I statistical error.

Whilst the current study offers original insights into the effects of UQH_2_ supplementation on mitochondrial respiration, and physiological and performance responses during severe-intensity exercise, there are some limitations. First, muscle and mitochondrial [CoQ_10_] were not measured, meaning it is not possible to confirm whether the observed improvements in mitochondrial oxidative phosphorylation efficiency were due to increased mitochondrial [CoQ10], or mediated by other mechanisms. In addition, mitochondrial ROS production, muscle lipid peroxidation markers and ANT1 + 2 and UCP-3 activation were not assessed and would have provided further insight into the mechanisms underpinning improved mitochondrial oxidative phosphorylation efficiency after UQH_2_ supplementation in the present study. Further research is required to resolve the mechanisms for lower mitochondrial CI_L_ respiration and improved oxidative phosphorylation coupling efficiency after UQH_2_ supplementation. Moreover, given that mitochondrial oxidative phosphorylation coupling efficiency is more likely to be compromised by prolonged endurance exercise and is accompanied by a loss in exercise economy in such settings (Fernström et al. 2007), further research is required to assess whether UQH_2_ supplementation may confer greater ergogenic potential for longer duration lower intensity exercise. It is also recognised that not pre-registering the trial publicly, and the possibility for inflated risk of type I statistical errors given then number of measures undertaken, are limitations of our study. The lack of female participants is also an important limitation of the current study which impedes external validity of our findings. Including women would likely have introduced additional biological- and hormonal-heterogeneity related to menstrual-cycle stage and/or hormonal contraceptive use, and the study was not powered to examine sex-by-treatment interactions or phase-specific responses. In that context, restricting enrolment to men was a design choice intended to preserve statistical precision for the primary outcomes rather than an assertion that women cannot be studied in this setting. Therefore, the results of the current study cannot be generalised to females and further research is required to assess the potential ergogenic and metabolic benefits of UQH_2_ supplementation in females.

In conclusion, short-term UQH_2_ supplementation increased plasma [CoQ_10_] and lowered CI_L_ respiration and 1/RCR suggesting improved mitochondrial oxidative phosphorylation coupling efficiency. These observations occurred independent of changes in skeletal muscle ANT1 + 2 and UCP-3 protein content as well as muscle CS activity and protein content of mitochondrial complexes I-V. However, UQH_2_ supplementation did not affect V̇O_2_ or T_Lim_ during severe-intensity exercise. Therefore, while short-term UQH_2_ supplementation improved skeletal muscle mitochondrial oxidative phosphorylation coupling efficiency, it did not translate to improved exercise economy, V̇O_2peak_ or T_Lim_ during severe-intensity cycling exercise in healthy, recreationally active adults.

## Data Availability

Data are available upon reasonable request.

## Abbreviations

ADP: Adenosine diphosphate
ANT1 + 2: Adenine nucleotide translocase1 + 2
BioPS: Biopsy preservation solution
CI–CIV: Mitochondrial protein complexes I–IV
CI_L_: Leak respiration through CI
CI_P_: ADP-stimulated respiration through CI
CI + II_P_: ADP-stimulated respiration through CI + II combined
CI + II_E_: ETS capacity through CI + II
CII_E_: ETS capacity through CII
CIV_E_: ETS capacity through CIV
CIV_res_: CIV reserve capacity
CoQ10: Coenzyme Q10
CS: Citrate synthase
E: Electron transfer system capacity
ETS: Mitochondrial electron transfer system
FCR: Respiratory flux control ratios
FOX-1: Ferrous oxidation in xylenol orange
GET: Gas exchange threshold
HDL: High-density lipoproteins
H_2_O_2_: Hydrogen peroxide
inv-RCR: Inverse respiratory control ratio
L: Leak respiration
LCR: Leak control ratio
LDL: Low-density lipoprotein cholesterol
LOOH: Lipid hydroperoxides
MIR05: MIR05 respiration medium
OXPHOS: Oxidative phosphorylation
O_2_: Oxygen
P: ADP-stimulated respiration
PCR: Phosphorylation control ratio
PLA: Placebo
ROS: Reactive oxygen species
SCR: Substrate control ratio
T_Lim_: Time to the limit of tolerance
UCP-3: Uncoupling protein-3
UQH_2_: Ubiquinol
V̇O_2_: Oxygen uptake
V̇O_2peak_: Peak oxygen uptake
V̇O_2_ SC: Oxygen uptake slow component
